# Are Internal or External Pancreatic Duct Stents the Preferred Choice for Patients Undergoing Pancreaticoduodenectomy? A Meta-Analysis

**DOI:** 10.1155/2017/1367238

**Published:** 2017-03-30

**Authors:** Yajie Zhao, Jianwei Zhang, Zhongmin Lan, Qinglong Jiang, Shuisheng Zhang, Yunmian Chu, Yingtai Chen, Chengfeng Wang

**Affiliations:** Department of Abdominal Surgical Oncology, National Cancer Center/Cancer Hospital, Chinese Academy of Medical Sciences and Peking Union Medical College, Beijing 100021, China

## Abstract

The technique of pancreatic duct stenting during pancreatic anastomosis can markedly reduce the incidence of postoperative pancreatic fistula (PF) after pancreaticoduodenectomy (PD). The method of drainage includes using either an external or an internal stent; the meta-analysis result shows us that there were no differences in the rates of postoperative complications between PD using internal stents and PD using external stents; internal stents may be more favorable during postoperative management of drainage tube. What is more, internal stents could reduce the digestive fluid loss and benefit the digestive function.

## 1. Introduction

Pancreaticoduodenectomy (PD) is the standard surgical procedure for pancreatic head tumors and periampullary region disease. Although advances in PD surgery and postoperative care have lowered the operative mortality rate to less than 5%, the surgical morbidity rate of PD is still high. About 35–60% of patients undergoing surgery will suffer from complications such as postoperative pancreatic fistula (POPF), delayed gastric emptying, and intra-abdominal collection [1]. Of these, POPF is one of the most important and common complications after PD; moreover, as high as 2–20% of patients die from POPF [2, 3]. Therefore, many surgical techniques have been undertaken and tested to prevent POPF and related complications, in order to lower the mortality rates. Despite some surgical strategies that have been shown to have a positive effect on decreasing POPF, including invagination anastomosis, duct-to-mucosa anastomosis [4, 5], and new style of digestive tract reconstruction, none of these methods can completely prevent POPF. In recent years, pancreatic drainage has been proven to eliminate the rate of POPF during pancreaticojejunostomy and decrease the risk of death after PD. Several meta-analyses of pancreatic drainage with internal and external stents have been published by many researchers. Most studies seemed to report the comparison of pancreatic drainage (with either internal or external stenting) and no pancreatic drainage during PD, with the former being associated with lower rates of POPF [6, 7]. However, to the best of our knowledge, a meta-analysis comparing the efficacy of internal and that of external drainage techniques has not yet been conducted. Therefore, this meta-analysis focused on comparing the postoperative mortality and complication rates between two methods of pancreatic drainage, in order to explore which drainage strategy is more effective and safe. The results of this meta-analysis could provide reliable evidence to give more guidance to those carrying out clinical work.

## 2. Materials and Methods

### 2.1. Search Strategy

We searched for journal articles published from January 2000 to September 2016 both electronically and manually. We searched the databases of PubMed, the Cochrane Library, Web of Science, EMBASE, China Biological Medicine, Chinese National Knowledge Infrastructure, Chinese Wangfang, and Chinese Science and Technology Periodicals for the following search terms: drainage, drainanges, drainaging, tent, stents, stenting, anastomosis, pancreatic resection, internal, external, in situ, ex situ, PD, PJ, pancreaticogastrostomy, Whipple, PF, pancreatic fistula, and pancreatic anastomosis. Both MeSH words and free terms were included in the search. No language restriction was applied and the search was performed by two independent researchers.

### 2.2. Inclusion Criteria

The following studies were included: (1) RCT studies; (2) those including patients with pancreatic head carcinoma or periampullary region disease who were treated with PD/PPPD; (3) those that reported pancreatic duct stenting following PD; (4) those that compared the incidences of postoperative complications between the internal and external stenting groups; and (5) those with complete data on the original complications.

### 2.3. Exclusion Criteria

The following studies were excluded: (1) non-RCT studies, (2) those in which the method of pancreaticojejunostomy anastomosis was not reported; (3) those that did not carry out a comparison between the internal and external stenting groups; (4) those which did not include postoperative complications and mortality rates as study outcomes; (5) repeated reports; (6) those with design flaws and low-quality studies; and (6) abstracts, case reports, letters, comments, and reviews without original data.

### 2.4. Literature Screening

Two independent investigators screened all the literature; when the two authors had a disagreement, they first tried to resolve it through discussion. If that failed, the final decision depended on a third person. EndNote reference management software was used to search and remove any duplicate studies.

### 2.5. Data Extraction

The following detailed data were independently extracted by the two investigators and checked by the other authors: title; authors; year of publication; country; study design; type of stents; surgery type; number of patients (age, sex); definitions and grade of PF; other postoperative complications such as delayed gastric emptying, intra-abdominal collections, and bile leak; and overall mortality rates.

### 2.6. Statistical Analysis

Review Manager (version 5.3.0) software provided by the Cochrane Collaboration was used to perform the meta-analysis in accordance with the PRISMA statement. Odds ratios (ORs) were used for the analyses of dichotomous variables and 95% confidence interval (CI) values were reported. The Mantel-Haenszel, Chi-square, and *I*^2^ tests were used to test the heterogeneity between included studies. If *I*^2^ < 50%, this suggested that the heterogeneity was not significant, and consequently a fixed effects model was used. If *I*^2^ > 50%, this suggested significant heterogeneity, and consequently a random effects model was applied. *P* < 0.05 was considered to be statistically significant. Funnel plots were used to assess any potential publication bias.

### 2.7. Characteristics of the Included Studies and Quality Assessment

On the basis of the inclusion and exclusion criteria, four randomized clinical trials were included in this meta-analysis. The total number of patients was 690, of whom 346 had external stenting and 344 had internal stenting. The detailed characteristics of all the included studies are shown in Figure 1 from [12] and Table 1. For more information, visit http://www.prisma-statement.org/. The quality of RCTs was evaluated based on the Jadad scale system, which was used to assess randomization, concealment of allocation, blinding, and withdrawals in the study. Each item was given a score of 0–2 and a score of 7 in total. If the total score was ≥5, the RCT was of high quality.

### 2.8. Assessment of Risk of Bias of Included RCTs

**Figure 2:**
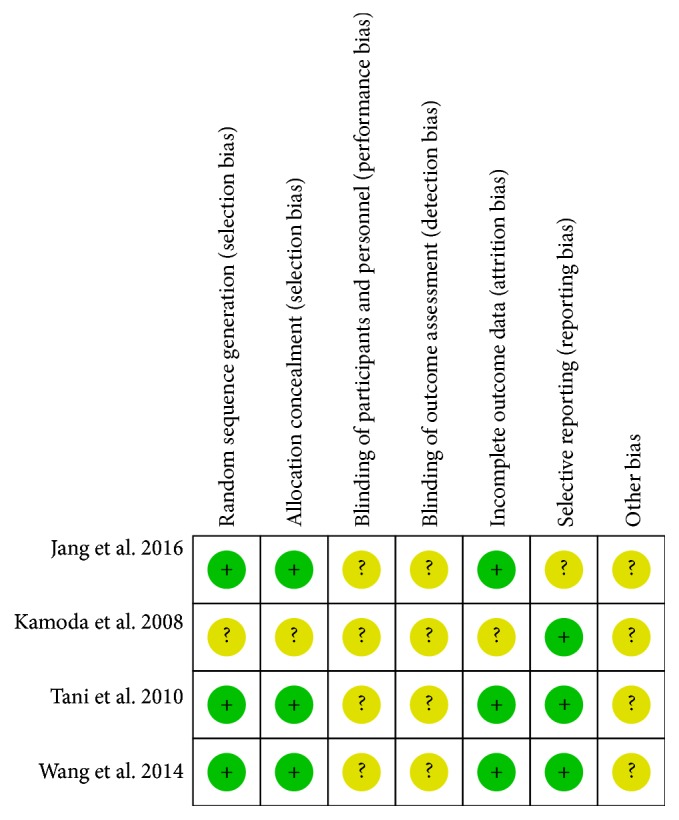


For the included RCTs, assessing the risk of bias included six aspects (allocation concealment, incomplete outcome data, blinding, selective reporting bias, sequence generation, and and other potential sources of bias) (Figure 2) by using the quality checklist recommended in the Cochrane Handbook. “Yes” indicated a low risk of bias, “Unclear” an uncertain risk of bias, and “No” a high risk of bias.

## 3. Meta-Analysis Results

### 3.1. Incidence of Pancreatic Fistula

**Figure 3:**
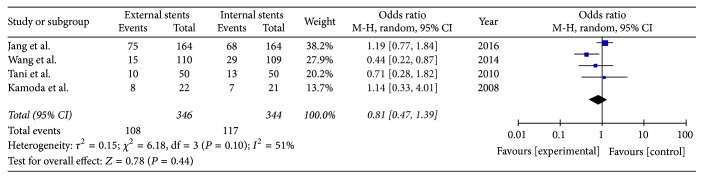
Meta-analysis of pancreatic fistula rate after pancreatic stenting (internal or external).

All four included studies reported the incidence of postoperative pancreatic fistula; we pooled data from the four studies to compare external stents group with internal stents group. The results of meta-analysis show that there is no difference between internal and external drainage in the rate of POPF (*I*^2^ = 51%, OR = 0.81; 95% CI, 0.47–1.39; *P* = 0.44); therefore the random model was used (Figure 3).

### 3.2. Incidence of Delayed Gastric Emptying

**Figure 4:**
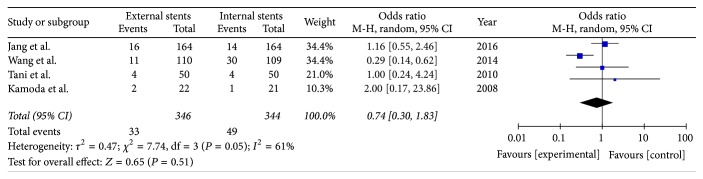
Meta-analysis of delayed gastric emptying after pancreatic stenting (internal or external).

Four studies reported the incidence of delayed gastric emptying after PD; there was a significant heterogeneity among studies (*I*^2^ = 61%); therefore the random model was used. The result (OR = 0.74; 95% CI, 0.30–1.83; *P* = 0.51) indicates that there was no statistical difference between two groups (Figure 4).

### 3.3. Incidence of Intra-Abdominal Fluid Collections

**Figure 5:**
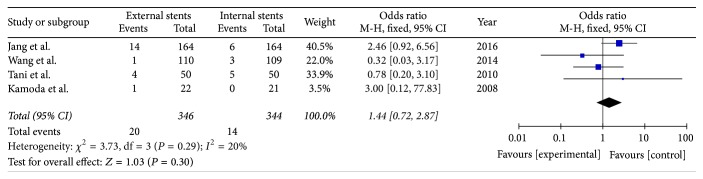
Meta-analysis of intra-abdominal fluid collections after pancreatic stenting (internal or external).

Four studies reported the incidence of intra-abdominal fluid collections (*I*^2^ = 20%) using a fixed model. The meta-analysis indicated that external drainage has no benefit compared to internal drainage in reducing the date of intra-abdominal fluid collections. There was no significant difference between the two methods (OR = 1.44; 95% CI, 0.72–2.87; *P* = 0.29) (Figure 5).

### 3.4. Incidence of Wound Infection

**Figure 6:**
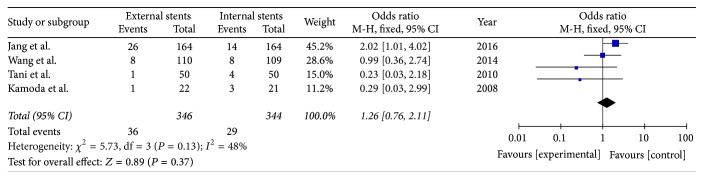
Meta-analysis of wound infection after pancreatic stenting (internal or external).

Four included studies reported the rate of wound infection after PD (*I*^2^ = 48%). *I*^2^ was 48% which revealed no obvious heterogeneity among these studies. Therefore, using a fixed model, there was no significant difference between external stents group and internal stents group resulting in wound infection (OR = 1.26; 95% CI, 0.76–2.11; *P* = 0.37) (Figure 6).

### 3.5. Bile Leak

**Figure 7:**
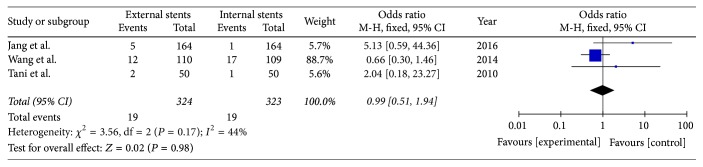
Meta-analysis of bile leak after pancreatic stenting (internal or external).

Three included studies reported the rate of bile leak after PD (*I*^2^ = 44%); fixed model was applied. There was no significant difference between external stents group and internal stents group (OR = 0.99; 95% CI, 0.51–1.94; *P* = 0.98) (Figure 7).

### 3.6. Abdominal Bleeding

**Figure 8:**
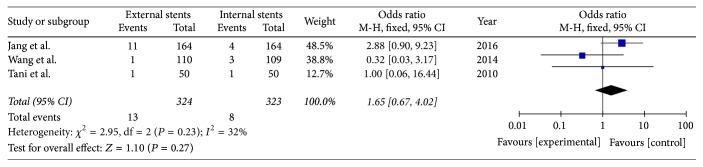
Meta-analysis of abdominal bleeding after pancreatic stenting (internal or external).

Three included studies reported the rate of abdominal bleeding after PD (*I*^2^ = 32%); fixed model was applied. There was no significant difference between external stents group and internal stents group (OR = 1.65; 95% CI, 0.67–4.02; *P* = 0.27) (Figure 8).

### 3.7. Gastrointestinal Bleeding

**Figure 9:**
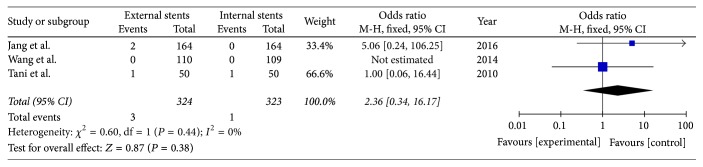
Meta-analysis of gastrointestinal bleeding after pancreatic stenting (internal or external).

Three included studies reported the rate of gastrointestinal bleeding after PD (*I*^2^ = 0%); fixed model was applied. There was no significant difference between external stents group and internal stents group (OR = 2.36; 95% CI, 0.34–16.17; *P* = 0.38) (Figure 9).

### 3.8. Overall Mortality

**Figure 10:**
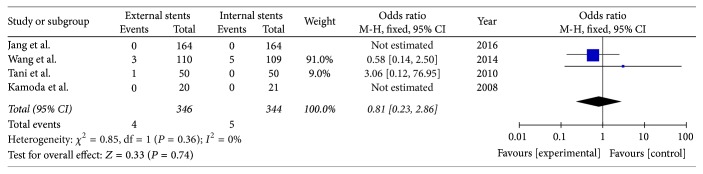
Meta-analysis of overall mortality after pancreatic stenting (internal or external).

Four included studies reported the rate of overall mortality after PD (*I*^2^ = 0%); fixed model was applied. There was no significant difference between external stents group and internal stents group (OR = 0.81; 95% CI, 0.23–2.86; *P* = 0.36) (Figure 10).

### 3.9. Subgroup Analysis of Pancreatic Fistula

Some studies reported that the texture of the pancreas and the method of anastomosis were independent risks factors for PF. Therefore, we classified the PF cases into three subgroups based on these above-mentioned parameters.

#### 3.9.1. Group Analysis Based on the Method of Anastomosis

**Figure 11:**
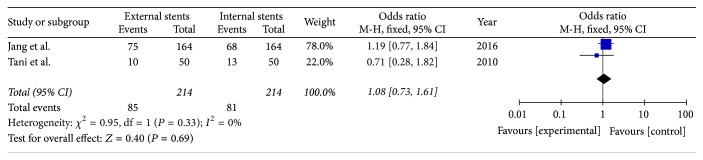
Meta-analysis of incidence of pancreatic fistula for patient undergoing end-to-side duct-to-mucosal anastomosis.

Two included studies reported the rate of PF for patients undergoing end-to-side duct-to-mucosal anastomosis (*I*^2^ = 0%). Fixed effects model was used, which revealed no significant differences between the internal and external stenting groups (OR = 1.08; 95% CI, 0.73–1.61; *P* = 0.69) (Figure 11).

#### 3.9.2. Group Analysis Based on the Texture of the Pancreas

**Figure 12:**
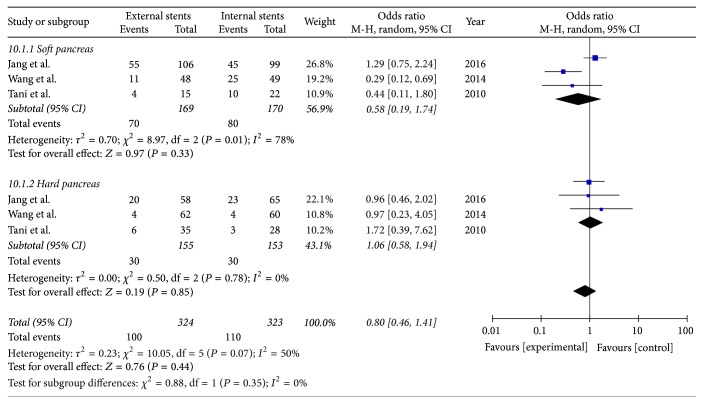
Meta-analysis of incidence of pancreatic fistula for patient with soft pancreas or hard pancreas.

Three studies reported POPF in patients with soft pancreas and hard pancreas. Using a random model revealed no statistically significant difference between the two stents of either soft pancreas subgroup or hard pancreas subgroup [soft pancreas subgroup (*I*^2^ = 80%, OR = 0.61; 95% CI 0.18–2.03; *P* = 0.86), hard pancreas subgroup (*I*^2^ = 0%, OR = 1.06; 95% CI 0.58–1.94; *P* = 0.85)] (Figure 12).

#### 3.9.3. Group Analysis Based on Severity of PF

**Figure 13:**
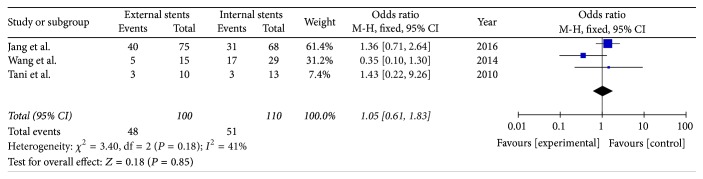
Meta-analysis of the severity of pancreatic fistula.

According to the International Study Group on Pancreatic Fistula (ISGPF) classification system, grade B/C fistulas require therapeutic interventions. The number of grade B and grade C fistulas among the PF patients can reflect the severity of PF. Our meta-analysis revealed no significant difference in the severity of PF between the two groups (*I*^2^ = 41%, OR = 1.05; 95% CI, 0.61–1.83; *P* = 0.85) (Figure 13).

### 3.10. Publication Bias

**Figure 14:**
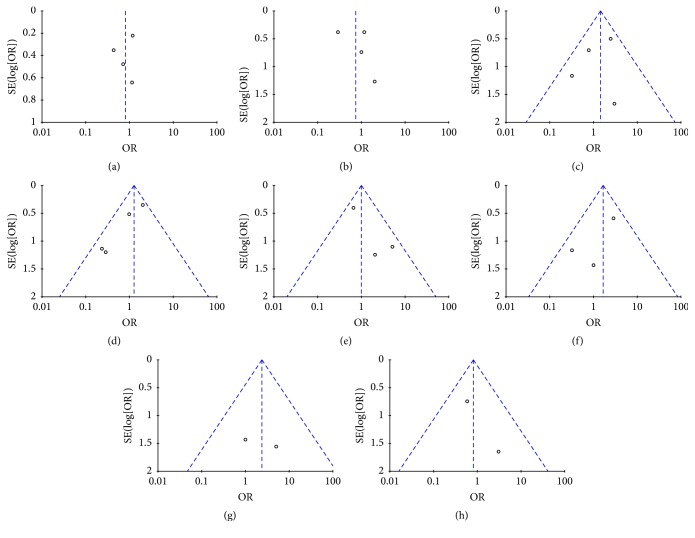
Funnel plots: (a) pancreatic fistula; (b) delayed gastric emptying; (c) intra-abdominal fluid collections; (d) wound infection; (e) bile leak; (f) abdominal bleeding; (g) gastrointestinal bleeding; (h) overall mortality.

**Figure 15:**
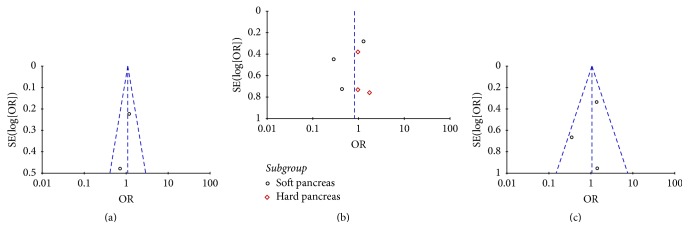
Subgroup funnel plots: (a) patient undergoing end-to-side duct-to-mucosal anastomosis; (b) patient with soft pancreas or hard pancreas; (c) severity of pancreatic fistula.

Funnel plots were conducted to assess the publication bias in this meta-analysis of included studies. As shown in Figures 14 and 15, there was no evident asymmetry in the funnel plots. Therefore, the result suggested a low probability of publication bias.

## 4. Discussion

Although surgical techniques and perioperative management have markedly decreased postoperative mortality associated with PD [6, 13], these reductions have not been accompanied by satisfactory improvements. Various complications have either direct or indirect relationships with PFs, such as intra-abdominal hemorrhage and abscess, which lead to prolonged hospital stays. Therefore, new technologies of anastomosis and reconstruction, such as pancreatic duct invagination anastomosis, have been used to increase the incidence of pancreatic leakage after PD. There are three main causes of POPF. First is the poor technology of anastomosis. Second, the pressure in the intestinal tube around anastomosed stoma boosts and influences the healing of pancreas-jejunum anastomotic stoma. Third, the pancreas-jejunum anastomotic stoma can be destroyed by leaking pancreatic juices and enzymes. Some RCTs and OCS studies have confirmed that drainage of the pancreatic duct using either an internal or an external stent can remarkably decrease the incidence of PF after PD. Although some surgeons prefer external stenting because of the short-term safety and stability after surgery, the long-term outcomes of this technique are duct dilatation and endocrine dysfunction. In addition, mechanical injury of the pancreatic duct may likely occur during stent removal, resulting in pancreatitis or tardive stenosis of the pancreatic duct. Therefore, some surgeons prefer internal drainage of the pancreatic duct using an internal stent in order to avoid drainage tube removal. However, external stents do have some advantages over an internal stent; for example, external stenting can prevent pancreatic-enzyme activation by bile and divert pancreatic secretion completely away from the anastomosis [14]. Some retrospective studies have reported a nil rate of PF by using the external stent technique. However, the efficacy and safety of drainage types in reducing and preventing postoperative complications are not yet completely understood. This meta-analysis concentrated mainly on discussing and evaluating effective and safe methods of stenting to reduce complications after PD [15–17].

Our results showed that neither internal nor external stents were associated with statistically significant differences in the incidence of PF. However, many factors such as the texture of the pancreas and pattern of anastomosis can affect the incidence of PF. The function of pancreatic exocrine for soft pancreas was normal and the secretion was high, resulting in a high incidence of PF [18]. To prevent various factors from affecting the meta-analysis results, we performed subgroup analyses based on the texture of the pancreas and pattern of anastomosis. The subgroup analyses result of three included RCT studies indicated that neither method had any obvious advantage or disadvantage in soft pancreas group and hard pancreas group. The optimum kind of anastomosis that can most effectively prevent PF has still not been completely approved. The end-to-side, duct-to-mucosa pancreaticojejunostomy; end-to-end, invagination pancreaticojejunostomy; and end-to-side invagination pancreaticojejunostomy are common and effective techniques. To decrease the heterogeneity that results from the different anastomotic methods, we carried out a subgroup analysis according to the pattern of pancreaticojejunostomy. Five included studies applied the end-to-side, duct-to-mucosa pancreaticojejunostomy during PD for all patients; however, the other four studies additionally applied the end-to-side invagination pancreaticojejunostomy. The outcome of this analysis revealed that dual drainage was not remarkably different in reducing PF for patients undergoing end-to-side, duct-to-mucosa pancreaticojejunostomy during PD.

The ISGPF classification system has become a useful criterion for characterizing clinical PF [19]. According to the ISGPF, we classified PF into three grades: A, B, and C. Grade B/C fistulas require therapeutic interventions. The duration of stay and rate and severity of complications are marginally increased and the costs are all higher for grade B/C than for grade A fistulas [20]. Our meta-analysis showed that external stents have no more benefit than internal stents in decreasing the severity of PFs.

Moreover, our results did not show any significant intergroup differences with respect to other complications. The postoperative complications are usually complex and multifaceted, and different complications can affect each other. For instance, the incidence of abdominal bleeding is associated with POPF and the incidence of delayed gastric emptying was also related to PF; thus, an effective drainage may only play a partial role in reducing and preventing the complication.

A major limitation was that we only included a small number of high-quality RCTs. The surgical experience and techniques used at different hospitals and care centers can produce different outcomes in PD and increase the heterogeneity between the included studies. Besides, the anastomotic technique of the surgeon, the texture of the remnant pancreas, the perioperative management, and the treatment of complications may affect the outcome of RCT that were included in this meta-analysis.

## 5. Conclusions

From this meta-analysis, no differences were found in postoperative outcomes between the two approaches. As internal stents may be more favorable during postoperative management of drainage tube, what is more, internal stents could reduce the digestive fluid loss and benefit to digestive function. Therefore, they could be the first choice in treating patients. Large multicenter RCTs are needed to better evaluate the most preferable pancreatic duct stenting strategies.

## Figures and Tables

**Figure 1:**
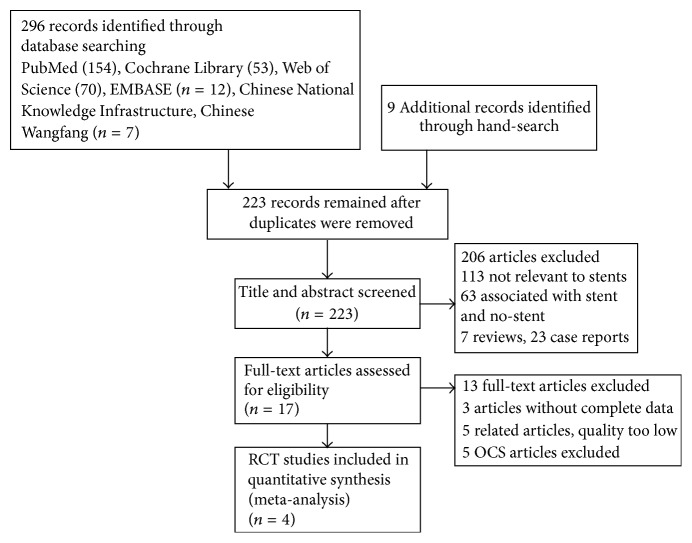


**Table 1:** The characteristics of all the included studies.

Author	Year	Country	Study type	Surgery	Group	Patients number	Male/female	Age, y	Anastomosis technique	Study quality RCT (Jadad system) Retro (NOS system)
Jang [8]	2016	Korea	RCT	PD/PPPD	EXS	164	103/61	62 (46.3–76.0)	ES-DM-PJ	5
					INS	164	87/77	63 (38.5–77.0)	ES-DM-PJ	
Wang [9]	2014	China	RCT	PD	EXS	110	59/51	52/58 (≥65/<65)	DM-PJ	5
					INS	159	54/55	56/53 (≥65/<65)	IN-PJ	
Tani [10]	2010	Japan	RCT	PD	EXS	50	28/22	70 (44–87)	ES-DM-PJ	5
					INS	50	27/23	68 (25–84)	ES-DM-PJ	
Kamoda [11]	2008	Japan	RCT	PD/PPPD	EXS	22	8/14	9/13 (≥65/<65)	ES-DM-PJ	5
					INS	21	7/14	14/7 (≥65/<65)	EE-IN-PJ	

RCT: randomized controlled trial; PD/PPPD: pancreaticoduodenectomy; EXS: external stents; INS: internal stents; ES-DM-PJ: end-to-side, duct-to-mucosa pancreaticojejunostomy; DM-PJ: duct-to-mucosa pancreaticojejunostomy; IN-PJ: invagination pancreaticojejunostomy; EE-IN-PJ: end-to-end, invagination pancreaticojejunostomy.
